# Self-harm and suicidality among three subgroups of male sex offenders: results from an Australian prisoner cohort

**DOI:** 10.1186/s40352-021-00146-6

**Published:** 2021-07-27

**Authors:** Mathew Gullotta, David Greenberg, Olayan Albalawi, Armita Adily, Azar Karminia, Lee Knight, Andrew Ellis, Tony Gerard Butler

**Affiliations:** 1 School of Population Health, University of New South Wales, Sydney, NSW 2052 Australia; 2 Justice Health and Forensic Mental Health Network, Sydney, NSW 2036 Australia; 3 School of Psychiatry, University of New South Wales, Sydney, NSW 2052 Australia; 4 Department of Statistics, Tabuk University, Tabuk, 47512 Saudi Arabia

**Keywords:** Sex offender, Epidemiology, Mental health problems, Self-harm, Suicide

## Abstract

**Objective:**

Prisoners complete suicide and self-harm more frequently than members of the community. Sex offenders have been found to be at greater risk of engaging in these behaviours. This study examines the characteristics, prevalence, and predictors of self-harm and suicide attempts among: sex offenders that only victimise children (ChildSOs); adults (AdultSOs); or both (age-crossover polymorphous; PolySOs).

**Methods:**

Data from three waves (1996, 2001, 2009) of the New South Wales (NSW) Inmate Health Survey was linked to the State’s re-offending database to identify men with histories of sexual offending. The health surveys captured self-report data on self-harm and suicidality.

**Results:**

Non-sexual violent offenders (15%) and AdultSOs (14%) had the highest rate of self-harm, significantly more than ChildSOs (11%), non-sexual non-violent offenders (10%), and PolySOs (0%). Several factors significantly predicted self-harm at the bivariate level for both ChildSOs and AdultSOs, with unique predictors for each group. At the multivariate level, manic-depression trended towards significance for ChildSOs and any mental health condition remained a significant predictor for AdultSOs who self-harmed relative to AdultSOs who had not (aOR = 11.989, 95%CI [1.14, 126.66]). Approximately 23% of AdultSOs, 22% of PolySOs, and 19% of ChildSOs reported a suicide attempt throughout their lifetime, whereas only 15% of non-sexual non-violent offenders reported an attempt. At the bivariate level, few factors were significant for ChildSOs while several factors were significant for AdultSOs. At the multivariate level, a diagnosis of depression and treatment with psychiatric medication trended towards being significant predictors of suicide attempts for ChildSOs. In contrast, treatment with psychiatric medication (aOR = 25.732, 95%CI [1.91, 347.19])] remained a significant predictor for AdultSOs who attempted suicide relative to AdultSOs who had not, as well as historical psychiatric hospitalisation (aOR = 6.818, 95%CI [1.04, 44.82]) and self-harm (aOR = 5.825, 95%CI [1.31, 25.99]).

**Conclusion:**

Sex offenders are at significantly higher risk of attempting and completing suicide relative to non-sexual non-violent offenders and warrant special attention. The prevalence rates and predictors of self-harm and suicidality suggest differences between sex offender subgroups may exist. These hold implications for the criminal justice and public health systems for addressing needs and identifying those most at risk of self-harm and suicide.

**Supplementary Information:**

The online version contains supplementary material available at 10.1186/s40352-021-00146-6.

## Introduction

High rates of suicide (Favril, Indig, et al., 2020) and self-harm (Favril, Yu, et al., 2020) are common among men in contact with the criminal justice system. Meta-analytic results suggest that up to 180 per 100,000 prisoners complete suicide (Fazel et al., 2017), approximately five to nine times that of people in the general community (Fazel et al., 2011). Furthermore, approximately 4 per 100 prisoners self-harm throughout their lives (Favril, Yu, et al., 2020).

Several offender groups appear to be more vulnerable to these acts. For example, a recent meta-analysis found that people with a sexual offence are approximately 3 times more likely to attempt suicide relative to offenders without these offences (Zhong et al., 2021). However, very little research has examined the prevalence and predictors of suicide attempts (e.g., Katsman & Jeglic, 2020) or self-harm (e.g., Stinson & Gonsalves, 2014) among sex offenders.

While society may have a negative view of sex offenders, particularly with regard to suicide and/or self-harm prevention and intervention programs for this group (Ayhan et al., 2017), clinical and research efforts should nonetheless be directed towards prevention in this group. From a pragmatic perspective, the economic and medical costs bore to society suggest they are worthwhile. In addition, the criminal justice system has responsibility for the health and safety of those under its authory. That is, there may be legal liability as well as legislative obligations that need to be upheld.

This article presents self-reported data of an Australian prisoner sample to describe the prevalence, characteristics, and factors associated with self-harm and suicidal behaviour among subgroups of sex offenders. Self-harm is defined as any self-injury or self-poisoning without the intention to die; and suicide attempts are defined as behaviours (self-injury or self-poisoning) with the intent to die (Victor & Klonsky, 2014).

## Literature review

Research on suicidal behaviour among prisoners with histories of sexual offences has primarily involved using official mortality records. Men charged with or convicted of sexual offences have been found to be over-represented in prison suicides in England and Wales (Dooley, 1990) and Scotland (Bogue & Power, 1995). A recent review of custodial deaths in England and Wales from 1978 to 2019 found prisoners convicted of sexual offences were the second highest offender group of all self-inflicted deaths (GOV.UK, 2021), despite representing a smaller proportion of the prison population. Statistical estimates suggested those convicted of a sexual offence were up to 24 times more likely to commit suicide than people without an offending history (Pritchard & Bagley, 2001) and approximately 5 times more likely to commit suicide compared to the general offender population (King et al., 2015).

Within group differences have also been observed. Men in Ireland under investigation for a sexual offence against a child were estimated to be 210 times more likely to commit suicide while in the community (1 person in 24) than their non-offending peers (1 in 5524); and men investigated for a sexual offence against an adult were estimated to be 3 times more likely (1 in 1644; Brophy, 2003). Higher rates were also found among men who only have sexual offences in their records than men with both sexual and non-sexual violent offences (Pritchard & King, 2004, 2005). It is possible that these rates underrepresent the true prevalence as data of completed suicides and official records are limited by the potential for misclassification of causes of deaths (e.g., accidental, natural, or unknown) or for underreporting by services.

While studies of completed suicides have indicated a vulnerability for sex offenders and different subgroups of sex offenders, few studies have examined suicide attempts. Reviews of archival records of sex offenders for suicide attempts found that approximately 14% (Jeglic et al., 2013) of incarcerated sex offenders attempted suicide at some point during their lives, and 39% of forensic psychiatric inpatient sex offenders attempted suicide (Stinson & Gonsalves, 2014). To our knowledge, there has only been one study that has used self-report data to describe suicide attempts in sex offenders. Katsman and Jeglic (2020) used data from a publicly available cross-sectional survey of inmates in the United States. Of 1118 men convicted of at least one sexual offence and surveyed, 18% had attempted suicide at some point throughout their life (Katsman & Jeglic, 2020). These authors also found no differences in the prevalence of suicide attempts between sex offender subgroups differentiated by victim age (Katsman & Jeglic, 2020; Stinson & Gonsalves, 2014).

In addition to prevalence rates, research has found that age, disrupted childhood environments (e.g., placement in out-of-home care), and a history of mental health problems including mood and anxiety disorders as well as personality disorders (e.g., borderline personality) were significant predictors for suicidality (Katsman & Jeglic, 2020; Stinson & Gonsalves, 2014). These appear to be similar to the key demographic (Bronson et al., 2017; Zhong et al., 2021) and mental health (Chang et al., 2015; Zhong et al., 2021) predictors of suicide attempts among the general prisoner population.

Another well-established predictor of suicide attempts is suicidal ideation (Fazel et al., 2008) which is common among sex offenders. Katsman and Jeglic (2020) also found that approximately 15% of sex offenders reported having thought about suicide at some point during their lives (Katsman & Jeglic, 2020). However, men who reported sexual attraction to minors were more likely to report such ideation. Of 333 adults in Israel who self-disclosed a sexual attraction to minors, many of whom had not offended nor been involved in the criminal justice system, almost 40% endorsed chronic suicidal ideation (Cohen et al., 2020).

In contrast to suicidal behaviours and ideation, self-harm behaviours do not involve the intent to end one’s life. A large cross-sectional survey of 14 prisons in the United Kingdom found that sex offenders were more likely to self-harm than their non-sexual offending peers (Liebling & Krarup, 1993). An audit of archival medical records in a forensic setting by Stinson and Gonsalves (2014) found that sex offenders were significantly more likely to have a history of self-harm than other inpatients (27% vs. 18%). Sex offenders who committed crimes involving both adults and children had higher rates of self-harm relative to sex offenders who offended against either adults or children only (30% vs. 23%). Mood disorders and borderline personality disorders predicted self-harm among the adult offenders and only impulse control disorders significantly predicted self-harm among the child offenders (Stinson & Gonsalves, 2014).

Scant research exists on the prevalence and predictors of: suicide attempts (Katsman & Jeglic, 2020) or self-harm (e.g., Stinson & Gonsalves, 2014), among subgroups of sex offenders differentiated by victim age. Most research findings have been based on examinations of archival or medical records, with few exploring self-reported histories. Archival and medical records may yield an underestimate of the prevalence rates for self-harm and suicidal behaviour as people who engage in these behaviours may not have required or sought medical attention (Jeglic et al., 2013). Such data sources may potentially only offer limited insights for prevention (Katsman & Jeglic, 2020). Using self-report data may assist in overcoming some of these concerns and provide more accurate lifetime prevalence rates.

Another gap in the current literature relates to the characteristics of the self-harm histories and suicide attempts of sex offenders. The method used, differences in the likelihood of these behaviours in the community and custody, and reasons for not completing the suicide attempt are valuable pieces of information that may inform intervention responses.

Lastly, there are contradictory results regarding the prevalence of suicidality between subgroups of sex offenders. Whereas men who committed sexual offences exclusively against children were found to be at heightened risk for completed suicide relative to those who committed sexual offences against adults (Brophy, 2003; Pritchard & King, 2005), there appeared to be no difference in terms of the prevalence of attempts between the groups (Katsman & Jeglic, 2020; Stinson & Gonsalves, 2014).

### The current study

This paper aimed to address these knowledge gaps by examining the self-reported histories of self-harm and suicide attempts among prisoners with and without histories of sexual offending. Sexual offenders were differentiated into subgroups of those whose convictions indicate they exclusively victimise children (child sex offenders (ChildSOs)); exclusively victimise adults or age-related peers (adult sex offenders (AdultSOs)); or those who victimise both (polymorphous or age-crossover sex offenders (PolySOs)). The prevalence of self-reported lifetime self-harm and suicide attempts, characteristics of these acts, and specific risk factors for ChildSOs, AdultSOs, and PolySOs were explored.

## Method

### Study design

This article presents data from a larger study which aimed to examine differences in the background histories and criminal careers (Gullotta et al., 2020) as well as the risk factors associated with recidivism (Gullotta et al., 2021) among subgroups of sex offenders. That study used a retrospective cohort design that involved data linkage of two data sources: (i) inmate health surveys and (ii) a reoffending database. The health surveys were cross-sectional in nature and only captured the most serious offence of the custodial sentence at the time of the survey. The linkage was performed to attain offending histories and identify those with historical sexual offences that were not captured by the surveys.

A full description of the data sources, the method for data linkage, and selection of the sample are described in detail elsewhere (Gullotta et al., 2020). Briefly, the health surveys were the 1996 (Butler, 1997), 2001 (Butler & Milner, 2003) and 2009 (Indig et al., 2010) New South Wales (NSW) Inmate Health Surveys. The cohort of participants in the NSW health surveys included random samples of prisoners from all adult correctional centres in NSW stratified by age, sex, and Indigenous status. The health surveys collected data on the physical health, mental health, and risk behaviours of this cohort. Only data from the men were retained for the larger study (*n* = 2114). Deterministic linkage using a unique identifier given to all prisoners in NSW, and in cases where this was not possible, common identifiers (name, sex, and date of birth), matched the cohort of participants from the surveys to the NSW Bureau of Crime Statistics and Research’s Re-offending Database (Hua & Fitzgerald, 2006). The reoffending database collected records on all finalised court matters in NSW between January 1994 and September 2014.^1^ Approximately 87% of the cohort could be linked to the reoffending database. The linkage allowed the cohort to be classified into groups based on types of offences, from the first available offence (provided by the reoffending database) until the offence at the time of participation in the survey.

Although this article presents secondary analyses of data from a larger study, the aims and analyses for this article are unique. Ethics approval for the linkage was provided by the Justice Health and Forensic Mental Health Network Human Research Ethics Committee (G70/14).

### Study cohort

The study cohort comprised of 2114 offenders overall. These offenders were then classified into: child sex offenders (ChildSOs) that included participants that committed sexual offences only against children (*n* = 77), adult sex offenders (AdultSOs) that included participants that committed sexual offences only against adults (*n* = 160), polymorphic or age-crossover sex offenders (PolySOs) that included participants that committed sexual offences against both children and adults (*n* = 43), as well as non-sexual violent offenders (*n* = 1269), and non-sexual non-violent offenders (*n* = 552).

### Measures

#### Outcome variables

There were two main outcome measures for this study. Self-harm was assessed by asking participants “excluding suicide attempts, have you ever deliberately harmed or injured yourself?” (0 = *no*, 1 = *yes*). Suicide attempts were assessed by asking participants if they had ever attempted suicide (0 = *no*, 1 = *yes*).

In order to provide a comprehensive description of self-harm and suicidality histories, subsequent variables on more specific details of the self-harm and suicide attempts were collected.

##### Self-harm

If the participant indicated they had self-harmed, they were subsequently asked the frequency (transformed into a binary categorical variable); whether they had ever self-harmed in prison; were more likely to self-harm in prison; and planned their self-harm prior to engaging in the behaviour. The method of self-harm was also assessed and transformed into a categorical variable (see Table 2).

##### Suicide attempts

If the participant indicated they had attempted suicide, they were subsequently asked: the frequency (numerical variable); whether they had: planned the suicide attempt; wanted to die when attempting; and the likelihood of attempting in prison. The method of the most recent suicide attempt^2^ was assessed and transformed into a categorical variable (see Table 4).  The reason for stopping the attempt was also assessed.

#### Correlates

The following potential correlates of self-harm and suicide attempts were included in the analysis based on findings from previous research (e.g., Favril, Yu, et al., 2020; Zhong et al., 2021). Age and Indigenous status were included as correlates. Other sociodemographic variables included any care placement as a child; completed less than high school education; working before prison; living in stable accommodation before prison; marital status; children; and whether it was their first time in custody. Suicidal ideation was included as a potential correlate and assessed by asking participants if they had ever *thought* about suicide and whether those thoughts had increased since entering prison. Other potential correlates also included clinical variables including previous diagnoses for a mental health disorder, assessed by asking participants if a doctor (or psychiatrist) had ever diagnosed any mental health disorder, and if yes, the condition or illness (see Table 1). Mental health treatment was determined from three separate questions covering: admission to a psychiatric facility (including unit or ward); ever prescribed psychiatric medication; or received treatment from a psychologist or counsellor.^3^

**Table 1:** Self-reported mental health status by offender group

	Total Sample (*n* = 2114)	ChildSO (*n* = 77)	AdultSO (*n* = 160)	PolySO (*n* = 43)	Violent (*n* = 1269)	Non-sexual non-violent (*n* = 552)	*X*^2^	*p* (Φ/ Φ_C_)
Sample members (%)	–	3.7%	7.6%	2.0%	60.4%	26.3%	–	–
Any mental health problem								
0 No	61.4%	48.7%	66.2%	60.5%	56.6%	73.2%	51.942***	<.001 (.16)
1 Yes	38.6%	51.3%	33.8%	39.5%	43.5%	26.8%		
ADD/ADHD								
0 No	93.9%	92.1%	94.7%	100%	92.2%	97.4%	20.919***	<.001 (.10)
1 Yes	6.1%	7.9%	5.3%	0	7.8%	2.6%		
Anxiety								
0 No	88.3%	84.2%	88.8%	88.4%	86.8%	82.3%	12.474*	.014 (.08)
1 Yes	11.7%	15.8%	11.2%	11.6%	13.2%	7.7%		
Depression								
0 No	70.3%	56.9%	68.1%	77.8%	69.9%	73.4$	7.052	1.33 (.07)
1 Yes	29.7%	43.1%	31.9%	22.2%	30.1%	26.6%		
Manic-depression								
0 No	93.9%	94.1%	94.5%	97.2%	92.7%	97.0%	8.322^+^	.080 (.08)
1 Yes	6.1%	5.9%	5.5%	2.8%	7.3%	3.0%		
Personality disorder								
0 No	95.3%	94.7%	94.7%	97.7%	94.1%	97.9%	12.832*	.012 (.01)
1 Yes	4.7%	5.3%	5.3%	2.3%	5.9%	2.1%		
Post-traumatic stress disorder								
0 No	98.9%	98.7%	98.7%	100%	99.0%	98.6%	1.163	.884 (.02)
1 Yes	1.1%	1.3%	1.3%	0	1.0%	1.4%		
Schizophrenia								
0 No	92.5%	88.2%	92,3%	100%	90.8%	97.7%	19.774**	.001 (.12)
1 Yes	7.5%	11.8%	7.7%	0	9.2%	2.3%		
Previous admission to psychiatric facility ^a^								
0 No	81.8%	80.6%	72.5%	81.0%	82.2%	84.1%	4.938	.294 (.08)
1 Yes	18.2%	19.4%	27.5%	19.0%	17.8%	15.9%		
Prescribed psychiatric medication								
0 No	86.9%	84.0%	86.4%	85.7%	84.6%	92.7%	21.621***	<.001 (.10)
1 Yes	13.1%	16.0%	13.6%	14.3%	15.4%	7.3%		
Psychological or counselling treatment								
0 No	66.9%	0.0%	67.4%	60.0%	66.8%	70.4%	8.349^+^	.080 (.08)
1 Yes	33.1%	50.0%	32.6%	40.0%	33.2%	29.6%		
Ever thought about suicide								
0 No	65.5%	61.3%	61.2%	54.8%	64.0%	71.7%	14.066**	.007 (.08)
1 Yes	34.5%	38.7%	38.8%	45.2%	36.0%	28.3%		
Suicidal thoughts increased since entering prison								
0 No	76.3%	66.7%	75.9%	63.6%	79.0%	71.5%	13.750^+^	.089 (.15)
1 Yes	23.7%	33.3%	24.1%	36.4%	21.0%	28.5%		

The information in this table is based the self-reported survey data. The sample size in this table reflect the number of participants who were able to be coded on each item. Some responses could not be coded because of the amount and quality of the information. The sample sizes ranged from ChildSOs: 31–77; AdultSOs: 69–160; PolySOs: 21–43; Violent: 523–1269; Non-sexual non-violent offenders: 232–552

^a^ Data from 1996 and 2001 surveys

^+^*p <* .10, ^*^ *p <* .05, ^**^p < .01, ^***^p < .001

### Statistical analysis

Descriptive statistics in self-reported self-harm and suicidality histories were presented, with differences between the sex-offender subgroups and non-sex offenders examined at the bivariate level (i.e., Chi-square tests for categorical variables and One-way Analysis of Variance and *Welch’s F* test for continuous variables). Effect sizes were interpreted as small (.20), medium (.50), and large (≥.60). Predictors of self-harm (outcome variable) for each specific sex offender subgroup were examined using bivariate logistic regression. Variables significant at the *p* ≤ 0.05 level were entered into a multivariate regression model. These analyses were also repeated for suicide attempts (outcome variable). Age was included in the models irrespective of statistical significance. This study adopted an exploratory approach to data analysis and as such an a priori alpha level of α = .05 for each statistical analysis was used. Statistical analyses were conducted using IBM® Statistical Package for the Social Sciences, version 23.

## Results

### Sample characteristics

Complete demographics of the study cohort are reported elsewhere (Gullotta et al., 2020). Briefly, PolySOs were the oldest of all the offender groups at 52 years of age on average, followed by ChildSOs that were 44 years, non-sexual non-violent offenders that were 37 years, AdultSOs that were 36 years, and lastly, violent offenders that were 31 years of age on average. AdultSOs (*n* = 67, 42%) had the largest proportion of participants who identified as Aboriginal or Torres Strait Islander, followed by violent offenders (*n* = 451, 36%), ChildSOs (*n* = 24, 31%), PolySOs (*n* = 11, 26%), and non-sexual non-violent offenders (*n* = 88, 16%).

The mental health status by offender type is presented in Table 1. ChildSOs were significantly more likely to report any mental health diagnosis, including anxiety, than the remaining groups. Diagnoses of ADD/ADHD, a personality disorder, and schizophrenia were significantly less common among the PolySOs and non-sexual non-violent offenders than the remaining groups. Significantly fewer non-sexual non-violent offenders had been prescribed psychiatric medication than the remaining groups. With regard to previous suicidal thoughts, PolySOs were significantly more likely to report such thoughts compared to the remaining groups. Similarly, both ChildSOs and AdultSOs were significantly more likely to report suicidal ideation compared to the non-sexual non-violent offenders. Slightly more ChildSOs and PolySOs reported an increase in suicidal ideation since entering custody compared to the remaining groups, although this difference was not significant.

### Characteristics of self-harm histories

Table 2 presents the prevalence of self-harm by offender type. Approximately 13% of the total sample reported a history of self-harm. AdultSOs (14%) and Violent offenders (15%) were significantly more likely to report a history of self-harm compared to non-sexual non-violent offenders (10%) as well as PolySOs (0%). The effect size of the differences between groups was small. The difference observed with the PolySOs may be an artefact of this groups small number as none in the current sample reported a history of self-harm.

**Table 2:** Self-harm histories by offender group

	Total Sample (*n* = 2114)	ChildSO (*n* = 77)	AdultSO (*n* = 160)	PolySO (*n* = 43)	Violent (*n* = 1269)	Non-sexual non-violent (*n* = 552)	*X*^2^	*p* (Φ/ Φ_C_)
Ever self-harmed								
0 No	86.8%	89.2%	85.7%	0	84.7%	90.3%	17.000**	.002 (.09)
1 Yes	13.2%	10.8%	14.3%		15.3%	9.7%		
Number of times							7.180^+^	.066 (.17)
1 Once	44.7%	57.1%	52.4%	–	39.3%	58.8%		
2 Twice or more	55.3%	42.9%	47.6%	–	60.7%	41.2%		
Self-harmed in prison								
0 No	71.5%	57.1%	71.4%	–	72.4%	70.6%	.798	.850 (.06)
1 Yes	28.5%	42.9%	28.6%		27.6%	29.4%		
Planned self-harm								
0 No	60.3%	57.1%	47.4%	–	61.6%	61.2%	1.496	.683 (.08)
1 Yes	39.7%	42.9%	52.6%		38.4%	38.8%		
Method of last self-harm							21.943*	.038 (.17)
1 Choke-inducing behaviours	3.1%	0	19.0%	–	1.7%	2.0%		
2 Impact with object	15.8%	14.3%	9.5%	–	16.7%	15.7%		
3 Overdose or poisoning	4.2%	0	0	–	4.4%	5.9%		
4 Use of an object	73.7%	85.7%	66.7%	–	74.4%	72.5%		
5 Other	3.1%	0	4.8%	–	2.8%	3.9%		
More likely to self-harm in prison than community								
0 No	69.0%	57.1%	71.4%	–	68.4%	71.4%	3.475	.747 (.12)
1 Yes	31.0%	42.9%	28.6%		31.6%	28.6%		

The information in this table is based the self-reported survey data. The sample size in this table reflect the number of participants who were able to be coded on each item. Some responses could not be coded because of the amount and quality of the information. The sample sizes ranged from ChildSOs: 31–77; AdultSOs: 69–160; PolySOs: 21–43; Violent: 523–1269; Non-sexual non-violent offenders: 232–552

^+^*p <* .10, ^*^ *p <* .05, ^**^p < .01

Of those that self-harmed, an equal proportion from each group reported having self-harmed: only once in their lifetime (total: 45%); when in prison (total: 29%); and/or after planning the act (total: 40%). Using an object (e.g., burning, self-amputation, slashing, or stabbing) was the most common method of the most recent self-harm for all offender groups, with ChildSOs (86%) significantly more likely to have used this method compared to the other groups. However, only a small effect size was observed for  this difference. An equal proportion from each group reported that they were more likely to self-harm in prison than in the community, although slightly more ChildSOs reported that they would.

### Correlates of self-harm

Table 3 presents the predictors of self-harm for ChildSOs and AdultSOs. For the ChildSOs, at the bivariate level, diagnosis of depression; manic depression or a personality disorder; treatment by psychiatric medication; and history of suicidal ideation, were significantly associated with increased odds of having self-harmed at some point throughout their lifetime. At the multivariate level, none of the variables remained significant.

**Table 3:** Bivariate and multivariate analyses for predictors of self-harm for ChildSOs and AdultSOs

	ChildSOs						AdultSOs					
	Bivariate			Multivariate			Bivariate			Multivariate		
	OR	95% CI	*p*	aOR	95% CI	*p*	OR	95% CI	*p*	aOR	95% CI	*p*
Age at survey	.947	[.98, 1.00]	.061	.951	[.88, 1.02]	.179	.958	[.92, 1.00]	.043	.930	[.86, 1.00]	.062
Aboriginal or Torres Strait Islander	.667	[.12, 3.58]	.636				2.400	[.94, 6.13]	.067			
Childhood care placement	.346	[.04, 3.01]	.336				1.146	[.39, 3.41]	.807			
Less than high school education	3.667	[.42, 31.73]	.238				3.173	[1.01, 9.98]	.048	1.685	[.33, 8.61]	.531
Employed before prison	.376	[.07, 2.00]	.252				1.000	[.40, 2.52]	1.000			
Unstable accommodation before prison	1.743	[.18, 17.13]	.634				.915	[.19, 4.38]	.911			
Single	1.640	[.38, 7.15]	.510				2.279	[.79, 6.61]	.130			
Has children	1.171	[.21, 6.42]	.856				1.224	[.39, 3.83]	.729			
First imprisonment	.704	[.15, 3.25]	.652				.507	[.17, 1.48]	.214			
Four or more chronic health conditions	1.667	[.37, 7.55]	.507				1.226	[.44, 3.43]	.698			
Any intravenous drug use	2.500	[.22, 27.94]	.457				2.786	[.94, 8.25]	.064			
Any mental health issue(s)	7.903	[.92, 67.87]	.060				4.632	[1.73, 12.39]	.002	11.989	[1.14, 126.66]	.039
ADD/ADHD	5.167	[.78, 34.30]	.089				4.033	[.89, 18.34]	.071			
Anxiety	3.800	[.77, 18.72]	.101				2.969	[.92, 9.54]	.068			
Depression	5.104	[1.10, 23.78]	.038	.375	[.03, 5.18]	.464	3.238	[1.18, 8.89]	.023	.084	[.01, 1.52]	.093
Manic-depression	21.333	[1.68, 271,.08]	.018	23.180	[.88, 613.36]	.060	4.444	[.69, 28.45]	.115			
Personality disorder	10.667	[1.27, 89.86]	.029	4.438	[.26, 75.01]	.302	2.105	[.40, 11.20]	.383			
Schizophrenia	5.083	[.77, 33.76]	.092				5.250	[1.08, 25.51]	.040	.628	[.00, 564.92]	.628
Previous admission to psychiatric facility^a^	2.875	[.21, 39.68]	.430				7.292	[2.25, 23.68]	.001	4.077	[.64, 26.10]	.138
Psychiatric medication	8.429	[1.72, 41.42]	.009	10.234	[.64, 163.14]	.100	4.346	[1.49, 12.72]	.007	7.861	[.45, 137.20]	.158
Psychological treatment	7.765	[.85, 70.75]	.069				2.790	[.85, 9.21]	.092			
Ever self-harmed	–	–	–	–	–	–	–	–	–	–	–	–
Thought about suicide	6.000	[1.12, 32.20]	.037	2.342	[.23, 24.12]	.475	9.137	[2.89, 28.92]	<.001	2.231	[.16, 31.53]	.553
Ever attempted suicide	4.333	[.83, 22.69]	.083				11.882	[4.19, 33.71]	<.001	4.861	[.44, 54.06]	.198

Adjusted odds ratios are presented with non-self-harming offenders of that group as the reference category. The sample sizes ranged from ChildSOs: 31–77 (final model Cox & Snell *R*^2^ = .209, Nagelkerke *R*^2^ = .415) and AdultSOs: 69–160 (final model Cox & Snell *R*^2^ = .246, Nagelkerke *R*^2^ = .469)

^a^ Data from 1996 and 2001 surveys

For the AdultSOs, at the bivariate level, age was significantly associated with a reduction in the likelihood of self-harm. Age; having left school with no qualification; any mental health issue including depression or schizophrenia; previous psychiatric treatment through an admission to a facility or by medication; suicidal ideations and attempts were significantly associated with increased odds of having self-harmed at some point throughout their lifetime. At the multivariate level, only age remained significant, with an increase in age resulting in approximately an 8% reduction (95% CI [.86, 1.0]) in the likelihood of self-harm. A previous diagnosis of any mental health condition was trending toward significance. Between 25% (Cox and Snell R^2^) and 47% (Naglekerke R^2^) of the variance was explained by the model.

### Characteristics of suicide attempts

Table 4 presents the prevalence of suicide attempts by offender type. Approximately one fifth of the study cohort reported attempting suicide, with significantly fewer non-sexual non-violent offenders reporting an attempt than the remaining groups. However, the effect size of this difference was small. AdultSOs who attempted suicide, reported an average of six attempts (*SD* = 19.08) which was significantly more than those that attempted from any of the remaining groups. Almost all AdultSOs that attempted suicide also indicated they had wanted to die, whereas far fewer ChildSOs felt the same way. Only a small effect size for this difference was observed.

**Table 4:** History of suicide attempts by offender group

	Total Sample (*n* = 2114)	ChildSO (*n* = 77)	AdultSO (*n* = 1650)	PolySO (*n* = 43)	Violent (*n* = 1269)	Non-sexual non-violent (*n* = 552)	*X*^2^	*p* (Φ/ Φ_C_)
Attempted suicide								
0 No	79.2%	81.2%	77.7%	78.1%	76.9%	84.8%	13.653**	.008 (.09)
1 Yes	20.8%	18.8%	22.3%	21.9%	23.1%	15.2%		
Number of attempts *M (SD*)	2.80 (11.94)	1.69 (2.78)	6.39 (19.08)	.38 (.65)	2.59 (11.12)	2.79 (12.70)	*wF*(4134.6) *=* 9.331***	<.001
Wanting to die when attempting suicide								
0 No	17.4%	41.7%	3.3%	28.6%	20.0%	9.2%	14.424**	.006 (.19)
1 Yes	82.6%	58.3%	96.7%	71.4%	80.0%	90.8%		
Planned suicide attempt								
0 No	38.2%	66.7%	28.6%	0	37.7%	43.5%	6.468	.167 (.14)
1 Yes	61.8%	33.3%	71.4%	100%	62.3%	56.5%		
Method of last suicide attempt^2^							15.861	.463 (.14)
1 Choke-inducing behaviours	39.3%	80.0%	40.0%	0	39.4%	36.0%		
2 Impact with object	10.7%	0	10.0%	0	11.7%	10.0%		
3 Overdose or poisoning	29.0%	20.0%	25.0%	100%	29.9%	26.0%		
4 Use of an object	8.4%	0	15.0%	0	5.1%	16.0%		
5 Other	12.6%	0	10%	0	13.9%	12.0%		
Reason for stopping							18.331	.566 (.21)
1 Change of heart	31.7%	0	25.0%	0	33.3%	38.9%		
2 Counselling	5.0%	0	0	0	7.6%	0		
3 Familial or partner concerns	39.6%	100%	41.7%	66.7%	37.9%	33.3%		
4 Physically stopped	8.9%	0	25.0%	33.3%	6.1%	5.6%		
5 Limited opportunity in jail	5.0%	0	8.3%	0	3.0%	11.1%		
6 Unspecified/ other	9.9%	0	0	0	12.1%	11.1%		
Likely or very likely to attempt in prison								
0 No	95.4%	90.2%	93.9%	95.7%	95.8%	95.6%	3.243	.518 (.05)
1 Yes	4.6%	9.8%	6.1%	4.3%	4.2%	4.4%		

The information in this table is based the self-reported survey data. The sample sizes ranged from ChildSOs: 31–77 and AdultSOs: 69–160.The sample size in this table reflect the number of participants who were able to be coded on each item. Some responses could not be coded because of the amount and quality of the information

^**^p < .01, ^***^p < .001

Of those that attempted suicide, there were no significant difference in terms of whether people had planned the attempt; nor the method used. The most commonly used method involved choke-inducing behaviours (40% on average), of which 91.4% involved hanging. A similar proportion of the groups reported a change of heart or familial and partner concerns as the reason for stopping the attempt. In terms of attempting suicide in prison, ChildSOs were slightly more likely to attempt whilst in prison than the remaining groups, however this difference was not statistically significant.

### Correlates of suicide attempts

Table 5 presents the predictors of suicide attempts for ChildSOs and AdultSOs. When examining predictors for suicide attempts for ChildSOs, only a history of depression and previous treatment with psychiatric medication were significant at the bivariate level. At the multivariate level, these variables were rendered non-significant; however, both variables were trending towards significance and accounted for between 24% (Cox and Snell R^2^) and 39% (Naglekerke R^2^) of the variance in suicide attempts.

**Table 5:** Bivariate and multivariate analyses for predictors of suicide attempts for ChildSOs and AdultSOs

	ChildSOs						AdultSOs					
	Bivariate			Multivariate			Bivariate			Multivariate		
	OR	95% CI	*p*	aOR	95% CI	*p*	OR	95% CI	*p*	aOR	95% CI	*p*
Age at survey	.970	[.92, 1.02]	.224	.964	[.91, 1.02]	.225	.971	[.93, 1.01]	.147	.957	[.91, 1.01]	.092
Aboriginal or Torres Strait Islander	1.810	[.47, 6.97]	.389				1.647	[.64, 4.22]	.298			
Sent to care as child	1.803	[.48, 6.81]	.384				1.925	[.70, 5.33]	.207			
Less than high school education	2.889	[.65, 12.80]	.163				1.400	[.54, 3.66]	.492			
Employed before prison	.260	[.06, 1.15]	.076				2.400	[.93, 6.23]	.072			
Unstable accommodation before prison	4.909	[.40, 59.85]	.212				1.098	[.23, 5.29]	.907			
Single	2.143	[.55, 8.39]	.274				1.786	[.67, 4.74]	.244			
Has Children	1.687	[.36, 7.88]	.506				2.187	[.79, 6.03]	.131			
First imprisonment	1.280	[.33, 4.94]	.720				.500	[.17, 1.49]	.212			
Four or more chronic health conditions	1.011	[.27, 3.78]	.987				1.892	[.64, 5.62]	.251			
Any intravenous drug use	.333	[.02, 5.03]	.427				.400	[.09, 1.80]	.232			
Any mental health issue(s)	1.111	[.24, 5.23]	.894				2.989	[1.15, 7.78]	.025	.269	[.03, 2.13]	.214
ADD/ADHD	1.091	[.17, 6.88]	.926				1.098	[.23, 5.29]	.907			
Anxiety	.455	[.08, 2.53]	.368				1.896	[.57, 6.31]	.297			
Depression	5.667	[1.25, 25.61]	.024	4.910	[.84, 28.79]	.078	2.444	[.86, 6.96]	.094			
Manic-depression	1.042	[.09, 12.66]	.974				1.593	[.21, 11.98]	.651			
Personality disorder	4.909	[.40, 59.85]	.212				.552	[.10, 3.05]	.495			
Schizophrenia	2.273	[.28, 18.27]	.440				9.167	[1.01, 83.05]	.049	.449	[.00, 791.47]	.834
Previous admission to psychiatric facility^a^	7.000	[.57, 86.32]	.129				6.643	[1.56, 28.35]	.011	6.818	[1.04, 44.82]	.046
Psychiatric medication	5.143	[1.13, 23.51]	.035	5.401	[.76, 38.61]	.093	13.579	[2.77, 66.68]	.001	25.732	[1.91, 347.19]	.014
Psychological treatment^a^	.467	[.10, 2.16]	.329				2.800	[.90, 8.72]	.076			
Ever self-harmed	4.333	[.32, 30.25]	.139				11.529	[2.94, 45.30]	<.001	5.825	[1.31, 25.99]	.021

Adjusted odds ratios are presented with non-self-harming offenders of that group as the reference category The sample sizes ranged from ChildSOs: 31–77 (final model Cox & Snell *R*^2^ = .242, Nagelkerke *R*^2^ = .386) and AdultSOs: 69–160 (final model Cox & Snell *R*^2^ = .228, Nagelkerke *R*^2^ = .371)

^a^ Data from 1996 and 2001 surveys

In terms of AdultSOs, at the bivariate level, any mental health issue including schizophrenia; previous psychiatric treatment through an admission to a facility or by medication; and a history of self-harm, were significant predictors of suicide attempts. At the multivariate level, previously psychiatric hospitalisation; psychiatric medication; and history of self-harm, remained significant predictors of suicide attempts in AdultSOs. AdultSOs who were previously hospitalised in a psychiatric facility were approximately 7 times (95% CI [1.04, 44.82]) more likely to attempt suicide than those who had never been hospitalised when holding constant age and other clinical variables. Similarly, AdultSOs treated by psychiatric medication were approximately 26 times (95% CI [1.9, 347.2]) more likely to attempt suicide than those who had not been treated by psychiatric medication, whilst holding all else constant. Similarly, AdultSOs who had a history of self-harm were 6 times (95% CI [1.3, 26.0]) more likely to attempt suicide than AdultSOs who did not have such a history, whilst holding all else constant. Between 23% (Cox and Snell R^2^) and 37% (Naglekerke R^2^) of the variance was explained by the model.

## Discussion

This article examined the prevalence and characteristics of self-reported lifetime self-harm (non-suicidal self-injurious or self-poisoning behaviour) and suicide attempts among male prisoners with and without a history of sexual offending recruited as a part of three waves of a comprehensive cross-sectional surveys of prisoners in NSW (Butler, 1997; Butler & Milner, 2003; Indig et al., 2010). Approximately 13% of the cohort reported a history of self-harm. These prevalence rates are less than that suggested by previous research which examined subgroups of sex offenders drawn from a psychiatric inpatient population (Stinson & Gonsalves, 2014), whereby histories of self-harm and suicide attempts are more likely to be observed. The random stratified samples recruited in the inmate health surveys reported in the current study provide valuable insights into the prevalence rates a representative sample of sex offenders that have served a custodial sentence in Australia.

A similar proportion of ChildSOs (11%) and AdultSOs (14%) reported histories of self-harm. This finding compliments previous research (Stinson & Gonsalves, 2014) which found no difference between the subgroups of sex offenders. However, none of the very few PolySOs sampled in the current study reported any history of self-harm. Although this finding contrasts with previous research which found PolySOs to be significantly more likely to report such histories relative to the ChildSOs and AdultSOs (Stinson & Gonsalves, 2014), this may reflect the small sample size of this group (*n* = 43) in the current study. Relative to the other sex offender subgroups, PolySOs appear to have different: demographic histories (Gullotta et al., 2020; Link & Lösel, 2021); personality patterns (e.g., Jackson & Richards, 2007); developmental perturbations (Lussier & Cale, 2013); and/or a generalised use of violence towards oneself and others. As such, it may be possible that PolySOs may have different prevalence rates of self-harm relative to ChildSOs and AdultSOs.

Of those from each group who reported a history of self-harm, there were similarities in the characteristics of their histories (e.g., frequency, level of planning, place, and likelihood of future self-harm whilst in custody). However, the groups differed with regard to the method used. ChildSOs were significantly more likely to use an object (e.g., burning, self-amputation, slashing, or stabbing) relative to the other sex offender groups and the non-sexual offenders. AdultSOs were the only group to endorse the use of choke-inducing behaviours (e.g., hanging, strangulation, or swallowing objects). The different methods indicate that some offender groups may be more likely to use more lethal methods, which holds clinical utility for: identifying and addressing the function of the behaviour; as well as intervention and management plans. These results are supportive of a general and ongoing need to screen all prisoners for self-harm on entry to custody and throughout their sentence.

Although the prevalence rates and background histories of self-harm were similar between the sex offender subgroups, ChildSOs appeared to have different predictors for self-harm relative to AdultSOs at the bivariate level. For the ChildSOs, a range of clinical variables and history of suicidal ideation were significant predictors for self-harm at the bivariate level. Unique to these offenders was self-reported previous diagnoses of either manic-depression or personality disorder. However, none of these variables remained as significant at the multivariate level. For the AdultSOs, age and educational attainment, along with other clinical variables and suicidal ideation and attempts, were found to be significant predictors for self-harm at the bivariate level. Educational attainment, any mental health diagnosis, schizophrenia, a previous admission to a psychiatric facility, and suicidal attempts were unique predictors to this offender group. Upon entering these at the multivariate level, only any previous mental health diagnosis remained a significant predictor. Depression was trending towards significance. The difference in clinical predictors of self-harm between the ChildSOs and AdultSOs is not surprising given the sex offender subgroups in the current study were found to have unique mental health profiles, a finding consistent with previous research (e.g., Ayhan et al., 2017; Dooley, 1990; Stinson & Gonsalves, 2014). Collectively, these findings suggest that there may be unique targets for assessment and intervention of self-harm behaviours among the different sex offender subgroups.

In terms of suicidality, approximately 21% of the sample reported that they had attempted suicide at some point throughout their lives. This finding is similar to that of international research of prisoners overall (e.g., 19% in New Zealand, Favril, Indig, et al., 2020; and 20% of Belgian prisoners, Favril et al., 2017). However, the prevalence of suicide attempts among our sample of sex offender subgroups was higher than the rate observed in other studies of sex offenders that used corroborated archival records (e.g., 14%, Jeglic et al., 2013) but consistent with that of studies using self-reported data (e.g., 19%, Katsman & Jeglic, 2020). It may be possible that self-reported data offers more accurate account of participants histories. However, the prevalence rate of the current study was smaller than that found in a sample of sex offenders from a psychiatric inpatient facility (Stinson & Gonsalves, 2014). Suicide attempts may occur more frequently in the clinical setting given the high likelihood of co-morbidities and severity of mental disorders.

Similar to previous research on suicide attempts (Jeglic et al., 2013; Katsman & Jeglic, 2020), this study found no significant difference in prevalence between the sex offender subgroups. However, the sex offender subgroups and non-sexual violent offenders were significantly more likely to report a history of suicide attempts compared to the non-sexual non-violent offenders. This finding supports the notion that some offender groups may be more vulnerable to these acts relative to others.

In observing the characteristics of the suicide attempts, significant differences were observed between the sex offender subgroups and their non-sexual offending peers. For example, AdultSOs had significantly more attempts on average than their child offending peers and the non-sexual offender groups. A significantly larger percentage of AdultSOs that attempted suicide also indicated that they wanted to die during the attempt. These findings contrast to the epidemiological studies that suggest sex offenders with child victims complete suicide at higher rates compared to those with adult victims (e.g., Brophy, 2003; Pritchard & King, 2004, 2005). The discrepancy between attempted and completed suicide for the sex offender subgroups may be due to several reasons. For example, it may be that ChildSOs use more lethal methods when attempting suicide than AdultSOs as there were a larger proportion of ChildSOs that reported using choke-inducing behaviours than AdultSOs, although this difference was not significant. It also appears that ChildSOs endorse fewer reasons for stopping the attempt relative to the AdultSOs. That is, AdultSOs may have more protective factors (or personally meaningful factors) that prevent them from completing suicide.

An alternative explanation is that people with sexual offences against children may not be captured by health surveys as they may be more likely to: think about, attempt, and subsequently complete suicide. PolySOs (45%) were significantly more likely to have reported lifetime suicidal ideation compared to the ChildSOs (39%) and AdultSOs (39%), and all subgroups were more likely to report lifetime suicidal ideation compared to the non-sexual non-violent offenders. While not statistically significant, a larger proportion of ChildSOs and PolySOs reported an increase in: suicidal thoughts since entering prison; and for ChildSOs, potential that they would attempt suicide whilst in prison. As such, sex offenders, particularly those with offences against children, may be at particular risk. These offenders may be vulnerable to: high levels of guilt and shame surrounding sexual offending (Marshall et al., 2009); practical concerns related to their reintegration into society, such as a loss of familial or support network, educational or career possibilities, and access to activities or services (Levenson & Hern, 2007); and/or stigmatisation as sexual offenders are openly targeted for severe forms of physical and psychological victimisation (Spencer, 2009), as well as sexual victimization (Man & Cronan, 2001). The policy and practical implications of these findings may involve improved training of suicide risk assessments for correctional staff, with particular attention drawn to ChildSOs and PolySOs at intake and throughout their custodial sentence, as well as to create a safe physical environment for sex offenders.

Bridging the gap between the prevalence rates and characteristics of the suicide attempt histories, risk factors for suicidality found potential differences. For the ChildSOs, a history of depression and treatment with psychiatric medication had marginal significance in predicting suicide attempts after controlling for age. In contrast, psychiatric treatment by admission and medication, as well as a history of self-harm were significant predictors of suicide attempts for AdultSOs. As such, these two groups had unique predictors. Clinical variables (e.g., psychiatric disorders) have been given significant prominence as risk factors for completed suicide among ChildSOs (Brophy, 2003). The presence of different mental health disorders predicted suicide attempts differently for ChildSOs and AdultSOs. However, research should also determine the predictive validity of the variance in psychiatric symptomology as well as the severity of the mental disorder. Taken together, it appears that there may be unique targets for assessment and intervention of suicide attempts among the different sex offender subgroups.

While examination of the sex offender subgroups differentiated by age is important, there was insufficient data to examine predictors of these behaviours for PolySOs. None of the PolySOs in our sample endorsed a history of self-harm which precluded the examination of predictors for this group. Additionally, very few of these offenders endorsed suicide attempts and this small number precluded a robust multivariate examination of predictors of suicidality. While such examinations could not be carried out in the current study, the description of the prevalence and characteristics of suicide attempts offers insight into this largely unknown group. From the available data, PolySOs represent a group that is unique from other sex offender subgroups and non-sex offenders (e.g., Stinson & Gonsalves, 2014). Future research should examine predictors of self-harm and suicidality among a sufficiently sized sample of sex offenders who have both children and adult victims.

### Limitations

This study has limitations which must be considered. Firstly, the current study presents a secondary analysis of existing data and as such is limited to the variables collected in the original health survey. More focused examinations of self-harm and suicide behaviours could not be conducted. Another limitation of this data concerns the cross-sectional nature of the health surveys. The current study is precluded from making causal inferences of risk factors. Future research may adopt prospective methodologies, particularly those with adolescent samples, to delineate associations between risk factors for suicide attempts and self-harm among specific offender types as well as examine temporality. The self-repot nature of the data may also have limitations. There may be response biases which result in under-reporting suicidal or self-harm behaviours. It is also possible that many mental health problems that were included as correlates were not assessed and diagnosed or had gone undetected (Butler, 1997) and therefore may reflect access to mental health professionals. Corroboration with clinical records would provide more accurate prevalence rates and predictor data. However, the results from this study can be considered a conservative estimate of the prevalence of these behaviours among the incarcerated sex offender subgroups.

## Conclusion

The prevalence of self-harm and suicidality (ideation and attempts) vary significantly between subgroups of sex offender and non-sex offenders. Given our study was limited to the custodial setting, the results presented are best representative of incarcerated offenders. Following the high prevalence of self-harm and suicide among prisoner populations compared to the general population, some offender types may be in need of intervention more than others. Sex offenders as a group are at significantly higher risk of attempting and completing suicide relative to non-sexual non-violent offenders and warrant special attention. This study highlights the need to provide adequate screening and intervention by correctional and mental health services for those who have come into contact with the criminal justice system for a sexual offence, with specific focus towards those with offences against child victims.

## Supplementary Information

**Additional file 1 **: **Table S1**. Bivariate and multivariate analyses for predictors of self-harm for non-sexual violent offenders and non-sexual non-violent offenders. **Table S2**. Bivariate and multivariate analyses for predictors of suicide attempts for non-sexual violent offenders and non-sexual non-violent offenders.

## Data Availability

The data generated and/or analysed during the current study are not publicly available as this was a condition of release of data to research by source data custodians.

## Abbreviations

AdultSOs: Refers to adult sex offenders or men who offended against adults only
ChildSOs: Refers to child sex offenders or men who sexually offended against children only
PolySOs: Refers to age-polymorphous sex offenders or men who offended against both children and adults

## Footnotes

1 After the original linkage, data for a cohort of participants (*n*=506) was collected from ROD and were available until July 2016.

2 Data were only available for the 1996 and 2001 inmate health surveys;

3 Data for this variable were only available for the 2001 and 2009 inmate health surveys.
