# Supplementary material for: Self-harm and suicidality among three subgroups of male sex offenders: results from an Australian prisoner cohort
Source: Health Justice. 2021 Jul 27;9:19. doi: 10.1186/s40352-021-00146-6 (PMC8317271; doi:10.1186/s40352-021-00146-6)
Supplement: Supplementary file 1 — Additional file 1 : Table S1. Bivariate and multivariate analyses for predictors of self-harm for non-sexual violent offenders and non-sexual non-violent offenders. Table S2. Bivariate and multivariate analyses for predictors of suicide attempts for non-sexual violent offenders and non-sexual non-violent offenders. [file 40352_2021_146_MOESM1_ESM.docx]

| Supplementary Table 1. Bivariate and multivariate analyses for predictors of self-harm for Non-sexual violent offenders and non-sexual non-violent offenders | | | | | | | | | | | | | | |
| --- | --- | --- | --- | --- | --- | --- | --- | --- | --- | --- | --- | --- | --- | --- |
|  | Non-sexual violent offenders | | | | | | | Non-sexual non-violent offenders | | | | | | |
|  | Bivariate | | | Multivariate | | | | Bivariate | | | | Multivariate | | |
|  | OR | 95% CI | *p* | aOR | 95% CI | *p* | OR | | 95% CI | *p* | aOR | | 95% CI | *p* |
| Age at survey | .969 | [.95, .99] | <.001 | .989 | [.93, 1.05] | .738 | .942 | | [.92, .97] | <.001 | .803 | | [.65, .99] | .042 |
| Aboriginal or Torres Strait Islander | 1.468 | [1.07, 2.02] | 0.18 | .710 | [.28, 1.79] | .466 | 2.178 | | [1.12, 4.23] | .021 | 5.852 | | [.21, 167.23] | .302 |
| Childhood care placement | 1.750 | [1.25, 2.44] | .001 | .850 | [.34, 2.15] | .731 | 3.091 | | [1.57, 6.09] | .001 | 1.605 | | [.16, 15.71] | .685 |
| Less than high school education | 1.351 | [.97, 1.89] | .078 | 2.947 | [1.05, 8.25] | .040 | 2.060 | | [1.10, 3.86] | .024 | .456 | | [.04, 5.33] | .531 |
| Employed before prison | .835 | [.61, 1.14] | .260 |  |  |  | .329 | | [.18, .62] | <.001 | .180 | | [.01, 2.45] | .198 |
| Unstable accommodation before prison | 1.646 | [1.07, 2.54] | .024 | .571 | [.16, 2.08] | .395 | 1.408 | | [.57, 3.50] | .461 |  | |  |  |
| Single | 1.455 | [1.03, 2.06] | .034 | .881 | [.31, 2.51] | .812 | 2.125 | | [1.14, 3.95] | .017 | .102 | | [.01, 1.13] | .062 |
| Has children | 1.143 | [.81, 1.60] | .442 |  |  |  | 1.492 | | [.70, 3.17] | .299 |  | |  |  |
| First imprisonment | .752 | [.53, 1.06] | .107 |  |  |  | .409 | | [.21, 82] | .012 | .120 | | [.00, 3.63] | .223 |
| Four or more chronic health conditions | 1.323 | [.87, 2.02] | .195 |  |  |  | 1.640 | | [.82, 3.27] | .160 |  | |  |  |
| Any intravenous drug use | 1.769 | [1.13, 2.77] | .013 | .312 | [.09, 1.03] | .056 | 2.491 | | [1.16, 5.36] | .020 | 1.046 | | [.07, 14.73] | .973 |
| Any mental health issue(s) | 2.793 | [2.02, 3.87] | <.001 | .275 | [.05, 1.63] | .155 | 4.267 | | [2.36, 7.71] | <.001 | .059 | | [.00, 101.96] | .456 |
| ADD/ADHD | 3.237 | [2.04, 5.14] | <.001 | 1.690 | [.27, 10.66] | .576 | 9.584 | | [3.09, 29.77] | <.001 | 20.321 | | [.03, 999.99] | .369 |
| Anxiety | 3.316 | [2.27, 4.84] | <.001 | .977 | [.17, 5.63] | .979 | 5.885 | | [2.81, 12.31] | <.001 | 390.217 | | [1.28, 999.99] | .041 |
| Depression | 3.277 | [2.36, 4.56] | <.001 | 3.135 | [.57, 17.11] | .187 | 5.038 | | [2.68, 9.48] | <.001 | .076 | | [.00, 4.70] | .221 |
| Manic-depression | 3.300 | [1.96, 5.55] | <.001 | 1.948 | [.28, 13.68] | .502 | 2.936 | | [.59, 14.54] | .187 |  | |  |  |
| Personality disorder | 3.418 | [2.05, 5.71] | <.001 | 1.206 | [.260, 5.59] | .811 | 2.259 | | [.47, 10.76] | .306 |  | |  |  |
| Schizophrenia | 3.002 | [1.86, 4.85] | <.001 | 2.680 | [.38, 19.15] | .326 | 4.128 | | [.78, 21.86] | .096 |  | |  |  |
| Previous admission to psychiatric facility^$^ | 3.440 | [2.09, 5.65] | <.001 | .518 | [.12, 2.32] | .390 | 6.554 | | [2.94, 14.63] | <.001 | .535 | | [.02, 12.62] | .698 |
| Psychiatric medication | 2.734 | [1.91, 3.92] | <.001 | .793 | [.17, 3.68] | .767 | 6.701 | | [2.91, 15.45] | <.001 | 5.030 | | [.09, 277.93] | .430 |
| Psychological treatment^$^ | 2.418 | [1.67, 3.51] | <.001 | .744 | [.18, 3.02] | .679 | 2.903 | | [1.25, 6.75] | .013 | 26.123 | | [.02, 999.99] | .379 |
| Ever self-harmed | --- | --- | --- | --- | --- | --- | --- | | --- | --- | --- | | --- | --- |
| Thought about suicide | 7.668 | [5.34, 11.01] | <.001 | 6.929 | [1.35, 35.63] | .021 | 8.643 | | [4.51, 16.57] | <.001 | 318.254 | | [2.21, 999.99] | .023 |
| Ever attempted suicide | 5.438 | [3.67, 8.05] | <.001 | 1.736 | [.43, 6.96] | .436 | 9.907 | | [4.51, 21.74] | <.001 | .141 | | [.00, 9.13] | .357 |
| *Note:* Adjusted odds ratios are presented with non-self-harming offenders of that group as the reference category. The sample sizes ranged from non-sexual violent offenders: 523-1,269 (final model Cox & Snell R^2^=.164, Nagelkerke R^2^=.271) and non-sexual non-violent offenders: 232-552 (final model Cox & Snell R^2^=.344, Nagelkerke R^2^=.699).  ^$^ Data from 1996 and 2001 surveys. | | | | | | | | | | | | | | |

| Supplementary Table 2. Bivariate and multivariate analyses for predictors of suicide attempts for Non-sexual violent offenders and non-sexual non-violent offenders | | | | | | | | | | | | | | |
| --- | --- | --- | --- | --- | --- | --- | --- | --- | --- | --- | --- | --- | --- | --- |
|  | Non-sexual violent offenders | | | | | | | Non-sexual non-violent offenders | | | | | | |
|  | Bivariate | | | Multivariate | | | | Bivariate | | | | Multivariate | | |
|  | OR | 95% CI | *p* | aOR | 95% CI | *p* | OR | | 95% CI | *p* | aOR | | 95% CI | *p* |
| Age at survey | .985 | [.97, 1.00] | .042 | .949 | [.91, .99] | .013 | .960 | | [.94, .98] | .001 | 1.019 | | [.96, 1.09] | .551 |
| Aboriginal or Torres Strait Islander | 1.187 | [.87, 1.62] | .281 |  |  |  | 2.698 | | [1.31, 5.56] | .007 | .622 | | [.11, 3.49] | .590 |
| Childhood care placement | 1.324 | [.96, 1.83] | .088 |  |  |  | 2.129 | | [1.01, 4.49] | .047 | 1.060 | | [.26, 4.39] | .936 |
| Less than high school education | 1.432 | [1.05, 1.96] | .024 | 1.176 | [.63, 2.20] | .611 | 2.319 | | [1.31, 4.11] | .004 | 1.221 | | [.33, 4.50] | .764 |
| Employed before prison | .879 | [.65, 1.19] | .401 |  |  |  | .463 | | [.26, .81] | .007 | .617 | | [.17, 2.25] | .465 |
| Unstable accommodation before prison | 1.555 | [1.02, 2.37] | .039 | 1.623 | [.72, 3.66] | .243 | 5.314 | | [2.27, 12.43] | <.001 | 2.422 | | [.42, 14.11] | .325 |
| Single | 1.371 | [1.00, 1.89] | .054 |  |  |  | 1.913 | | [1.09, 3.35] | .023 | 1.045 | | [.25, 4.34] | .952 |
| Has children | 2.602 | 1.87, 3.63] | <.001 | 2.200 | [1.18, 4.10] | .013 | 1.772 | | [.03, 3.38] | .082 |  | |  |  |
| First imprisonment | .832 | [.60, 1.15] | .267 |  |  |  | .306 | | [.16, .58] | <.001 | .668 | | [.19, 2.42] | .539 |
| Four or more chronic health conditions | 1.854 | [1.22, 2.82] | .004 | 1.656 | [.71, 3.89] | .247 | 1.025 | | [.52, 2.01] | .944 |  | |  |  |
| Any intravenous drug use | .487 | [.27, .88] | .017 | 2.141 | [1.05, 4.35] | .035 | 1.104 | | [.50, 2.43] | .806 |  | |  |  |
| Any mental health issue(s) | 3.488 | [2.53, 4.80] | <.001 | 2.615 | [1.01, 6.74] | .047 | 3.772 | | [2.12, 6.70] | <.001 | 3.026 | | [.47, 19.32] | .242 |
| ADD/ADHD | 1.243 | [.78, 1.99] | .366 |  |  |  | 3.326 | | [.91, 12.16] | .069 |  | |  |  |
| Anxiety | 1.528 | [1.05, 2.22] | .026 | 1.089 | [.32, 3.66] | .891 | 2.142 | | [.97, 4.71] | .058 |  | |  |  |
| Depression | 2.624 | [1.90, 3.62] | <.001 | 2.321 | [.92, 5.86] | .075 | 3.076 | | [1.64, 5.76] | <.001 | 2.216 | | [.50, 9.84] | .295 |
| Manic-depression | 3.327 | [1.93, 5.72] | <.001 | 5.279 | [.95, 29.28] | .057 | 2.739 | | [.71, 10.52] | .142 |  | |  |  |
| Personality disorder | 2.066 | [1.24, 3.45] | .005 | 4.267 | [.97, 18.76] | .055 | 2.111 | | [.42, 10.72] | .367 |  | |  |  |
| Schizophrenia | 2.562 | [1.57, 4.18] | <.001 | 1.862 | [.49, 7.15] | .365 | 2.143 | | [.42, 10.89] | .358 |  | |  |  |
| Previous admission to psychiatric facility^$^ | 12.640 | [5.81, 27.51] | <.001 | 1.041 | [.39, 2.76] | .935 | 31.167 | | [7.03, 138.24] | <.001 | 6.120 | | [1.16, 32.25] | .033 |
| Psychiatric medication | 2.731 | [1.87, 3.98] | <.001 | 1.116 | [.43, 2.93] | .824 | 2.254 | | [.99, 5.14] | .053 |  | |  |  |
| Psychological treatment^$^ | 3.941 | [2.76, 5.63] | <.001 | .851 | [.36, 2.00] | .711 | 10.306 | | [4.46, 23.81] | <.001 | 1.455 | | [.26, 8.05] | .667 |
| Ever self-harmed | 5.438 | [3.67, 8.05] | <.001 | 4.046 | [1.86, 8.79] | <.001 | 9.907 | | [4.51, 21.74] | <.001 | 6.658 | | [1.32, 33.60] | .022 |
| *Note:* Adjusted odds ratios are presented with non-self-harming offenders of that group as the reference category. The sample sizes ranged from non-sexual violent offenders: 523-1,269 (final model Cox & Snell R^2^=.228, Nagelkerke R^2^=.350) and non-sexual non-violent offenders: 232-552 (final model Cox & Snell R^2^=.240, Nagelkerke R^2^=.450).  ^$^ Data from 1996 and 2001 surveys. | | | | | | | | | | | | | | |
